# Intravenous overload of fluids and sodium may contribute to the lower infusion of enteral nutrition in critically ill patients

**DOI:** 10.5935/0103-507X.20190032

**Published:** 2019

**Authors:** Diana Borges Dock-Nascimento, Suzana Souza Arantes, João Manoel Silva Jr, José Eduardo de Aguilar-Nascimento

**Affiliations:** 1 Departamento de Alimentos e Nutrição, Faculdade de Nutrição, Universidade Federal de Mato Grosso - Cuiabá (MT), Brasil.; 2 Programa de Pós-Graduação em Ciências da Saúde, Universidade Federal de Mato Grosso - Cuiabá (MT), Brasil.; 3 Curso de Nutrição, Centro Universitário de Várzea Grande - Várzea Grande (MT), Brasil.; 4 Programa de Pós-Graduação em Anestesiologia, Universidade São Paulo - São Paulo (SP), Brasil.; 5 Departamento de Anestesiologia, Hospital do Servidor Público Estadual "Francisco Morato de Oliveira" - São Paulo (SP), Brasil.; 6 Curso de Medicina, Centro Universitário de Várzea Grande - Várzea Grande (MT), Brasil.

**Keywords:** Critical care, Fluids, Sodium, Enteral nutrition, Cuidados intensivos, Fluido, Sódio, Nutrição enteral

## Abstract

**Objective:**

To evaluate the effects of intravenous infusion of fluids and sodium on the first day of admission on infusion of enteral nutrition in the first 5 days in intensive care patients.

**Methods:**

A prospective cohort study was conducted with critical nonsurgical patients admitted for at least 5 days who were on mechanical ventilation and receiving enteral nutrition. The amount of intravenous fluids and sodium infused on the first day and the volume of enteral nutrition infused in the first 5 days were investigated. The volume of intravenous fluids > 35mL/kg or ≤ 35mL/kg of body weight and sodium (above or below the 25th percentile) infused on the first day was compared with infused enteral nutrition.

**Results:**

A total of 86 patients were studied, with a mean (± standard deviation) of 65 ± 17 years, of which 54.7% were female. On the first day, 3,393.7 ± 1,417.0mL of fluid (48.2 ± 23.0mL/kg) and 12.2 ± 5.1g of sodium were administered. Fifty-eight (67.4%) patients received more than 35mL/kg of fluids. In 5 days, 67 ± 19.8% (2,993.8 ± 1,324.4mL) of the prescribed enteral nutrition was received. Patients who received > 35mL/kg of intravenous fluids also received less enteral nutrition in 5 days (2,781.4 ± 1,337.9 *versus* 3,433.6 ± 1,202.2mL; p = 0.03) *versus* those who received ≤ 35mL/kg. Patients with intravenous sodium infusion above the 25th percentile (≥ 8.73g) on the first day received less enteral nutrition volume in 5 days (2,827.2 ± 1,398.0 *versus* 3,509.3 ± 911.9mL; p = 0.02).

**Conclusion:**

The results of this study support the assumption that the administration of intravenous fluids > 35mL/kg and sodium ≥ 8.73g on the first day of hospitalization may contribute to the lower infusion of enteral nutrition in critically ill patients.

## Abstract (pt)

**Objetivo:**

Avaliar os efeitos da administração intravenosa de fluidos e sódio no primeiro dia de internação com a infusão de nutrição enteral em pacientes de terapia intensiva.

**Métodos:**

Estudo de coorte prospectivo realizado com pacientes críticos, não cirúrgicos, em ventilação mecânica internados pelo menos há 5 dias com nutrição enteral. Investigaram-se a quantidade de fluidos e sódio administrados por via venosa no primeiro dia e o volume de nutrição enteral infundido nos primeiros 5 dias. Comparou-se o volume de fluidos intravenosos do primeiro dia > 35mL/kg ou ≤ 35mL/kg de peso corporal e de sódio (acima ou abaixo do percentil 25), com o total de nutrição enteral infundida.

**Resultados:**

Estudaram-se 86 pacientes com média (± desvio padrão) de 65 ± 17 anos, sendo 54,7% do sexo feminino. Foram administrados, no primeiro dia, 3.393,7 ± 1.417,0mL de fluidos (48,2 ± 23,0mL/kg) e 12,2 ± 5,1g de sódio. Cinquenta e oito (67,4%) pacientes receberam mais de 35mL/kg de fluidos. Em 5 dias, foram ofertados 67 ± 19,8% (2.993,8 ± 1.324,4mL) da nutrição enteral. Os pacientes que receberam > 35mL/kg de fluidos intravenosos também receberam menos nutrição enteral em 5 dias (2.781,4 ± 1.337,9 *versus* 3.433,6 ± 1.202,2mL; p = 0,03) *versus* quem recebeu ≤ 35mL/kg. Pacientes com infusão de sódio intravenoso acima do percentil 25 (≥ 8,73g) no primeiro dia receberam menos volume de nutrição enteral em 5 dias (2.827,2 ± 1.398,0 *versus* 3.509,3 ± 911,9mL; p = 0,02).

**Conclusão:**

Os resultados deste estudo apoiam o pressuposto de que a administração de fluidos intravenosos no primeiro dia de internação > 35mL/kg e de sódio ≥ 8,73g pode contribuir para a menor infusão de nutrição enteral em pacientes críticos.

## INTRODUCTION

Resuscitation with fluid infusion is one of the most frequent interventions performed in patients in intensive care, especially in the presence of shock.^(1)^ The physiological aim of resuscitation is to restore or maintain the effective circulating volume to ensure adequate tissue perfusion.^(2-4)^ However, excess fluid can result in adverse effects.^(5,6)^ Rapid redistribution of the infused volume leads to capillary damage^(7)^ and an increase in capillary permeability; thus, only 5 to 20% of infusions remain intravascular 90 minutes after infusion.^(8)^ This excess also results in sodium and water retention, with consequent acute kidney injury and impaired oxygen supply to tissues due to the low effective circulating volume.^(9)^ In this context, several studies have shown increased mortality with intravenous fluid overload.^(10-13)^ Thus, more restrictive fluid administration strategies may be beneficial to avoid overload and increased morbidity and mortality,^(10,14,15)^ especially on the first day, when the patient receives more volume. It is important to note that the terms "restrictive" and "liberal" in fluid administration are not uniform.^(10)^ On the other hand, there is a close relationship between nutritional therapy and fluid supply. In clinical practice, nutrients, water, and electrolyte balance are interconnected during treatment,^(16)^ and calories and nutrient supply are crucial for reducing complications and death.^(17,18)^ However, in clinical practice, only 50 to 87% of the prescribed enteral diet is effectively infused.^(19,20)^ In this relationship between nutrients and fluids, a high infusion of crystalloid solutions results in anasarca, inadequate weight gain,^(21)^ intestinal loop edema, gastroparesis, vomiting, and adynamic ileus. These adverse effects, caused by an excess volume of fluids and sodium, may contribute to lower administration of an enteral diet and an increase in caloric and protein deficits.^(18)^ Thus, the guidelines of the Surviving Sepsis Campaign^(22)^ recommend that patients with hypoperfusion, hypotension or hypovolemia receive an initial volume of fluids of only 30mL/kg. Corroborating this recommendation, the consensus Guidelines on Intravenous Fluid Therapy for Adult Surgical Patients (GIFTASUP) also recommend a restrictive intravenous fluid volume.^(23)^

This study aimed to evaluate the effects of intravenous administration of fluids and sodium on the first day of hospitalization with the infusion of an enteral diet in nonsurgical intensive care patients undergoing mechanical ventilation.

## METHODS

A prospective cohort study was conducted between October 2014 and December 2015 in the intensive care unit (ICU) of a private hospital (*Hospital Santa Rosa*) in Cuiabá (MT). The study was approved by the Ethics Committee on Research with Humans (CAAE: 37465414.0.0000.5541) and was conducted in accordance with the Declaration of Helsinki (2000). Relatives or guardians of patients signed an informed consent form (ICF).

Included patients were admitted to the ICU for at least 5 days for clinical, nonsurgical, mechanical ventilation during the first 24 hours of hospitalization and received exclusive enteral nutritional therapy (ENT). Surgical patients undergoing spontaneous breathing, pregnant women, those with late onset of nutrition (> 48 hours of hospitalization), those who received exclusive parenteral or enteral-associated nutrition, those with hemodynamic instability, and those who died within the first 5 days of hospitalization were excluded.

The outcome variables investigated were the total volume of intravenous fluids administered on the first day of hospitalization (mL) in mL/kg of body weight, grams of sodium administered on the first day of hospitalization, total volume of ENT prescribed and infused in 5 days, volume of ENT prescribed and infused on the first day, the percentage of ENT infused in 5 days (enteral feeding volume infused in 5 consecutive days × 100/total volume of ENT prescribed in 5 days) and the protein-calorie deficit over 5 days. Considering the recommendations regarding fluid resuscitation in the acute phase^(22,23)^ and according to the ASPEN Board of Directors and the Clinical Guidelines Task,^(24)^ which recommends 30 to 40mL/kg of body weight, the volume of intravenous fluids was categorized as > 35mL/kg/day and ≤ 35mL/kg/day. For statistical analysis, the sodium value administered was categorized below or above the 25th percentile (8.73g) because this was the lowest interquartile range. The volume of intravenous fluids infused on the first day/kg of body weight (≤ 35mL/kg or > 35mL/kg) and the amount of sodium above or below the 25th percentile were correlated with the total enteral diet prescribed and infused in 5 days (mL), the volume of ENT prescribed and infused on the first day, the percentage of prescribed enteral diet infused, and the protein-calorie deficiency over 5 days.

To characterize the sample, age, patients ≥ 60 years, sex, estimated body weight, main causes that led to hospitalization, most frequent digestive tract disorders (constipation, diarrhea, abdominal distention, and melena), volume drained by nasogastric tube (NG tube; for patients who required this procedure), Simplified Acute Physiology Score III (SAPS III) score, amount of noradrenaline (mcg/kg/minute), biochemical measurements (mean of 5 days of collection) of C-reactive protein (CRP; mg/L), serum albumin (g/dL), lactate (mmol/L) and serum glucose (mg/dL) were recorded, and the CRP/albumin ratio was calculated. The nutritional status of patients in the first 24 hours of admission and their calorie and protein requirements were also evaluated. The length of stay in the ICU and ICU mortality at 28 days were also recorded. Assessment of nutritional status was performed by the subjective global assessment (SGA A corresponded to well-nourished; SGA B, corresponded to risk of malnutrition or moderate malnutrition, and SGA C, corresponded to severe malnutrition).

### Protocol of nutritional therapy

Enteral diet was started in the first 24 hours but only in the presence of hemodynamic stability and after confirmation of the location of the probe by X-ray. Calorie and protein requirements were assessed according to the European Society for Parenteral and Enteral Nutrition (ESPEN).^(17)^ A total of 25 to 30kcal/kg and 1.25 to 2.0g of protein/kg of body weight were calculated. It was planned to reach the calculated need on the third or fourth day of diet. To achieve this goal, a third and a quarter of the need/day of enteral nutrition were prescribed.

### Volume of intravenous fluids and sodium

During the first 5 days of hospitalization, the volume of intravenous fluids and the amount of sodium administered were recorded. Crystalloids (0.9% saline solution; simple Ringer's solution, lactated Ringer's solution or glycoprotein solution), colloids, distilled water, dilution serum, drug volumes, and, lastly, blood or derivatives were considered for this purpose. The amount of sodium (grams) was determined according to the amount of crystalloid fluid administered on the first day, according to the composition of the saline solution. The researcher had no influence on the choice or method of fluid resuscitation, which was performed as the intensive care physician judged necessary.

### Statistical analysis

The chi-square test was used for the categorical variables. Continuous variables were analyzed by Levene's test to ascertain homogeneity, followed by the Kolmogorov-Smirnov test to determine normality. Student's t-test was used for homogeneous data with a normal distribution. Nonhomogeneous data were analyzed using the nonparametric Mann-Whitney test. The volume of infused enteral therapy (< or ≥ 2,063mL) was categorized over 5 days by the 25th percentile. Continuous data are expressed as the mean ± standard deviation or median and variation. A significance level of 5% (p < 0.05) was established. Statistical Package for the Social Sciences (SPSS) version 20.0 was used.

## RESULTS

**Figure 1:**
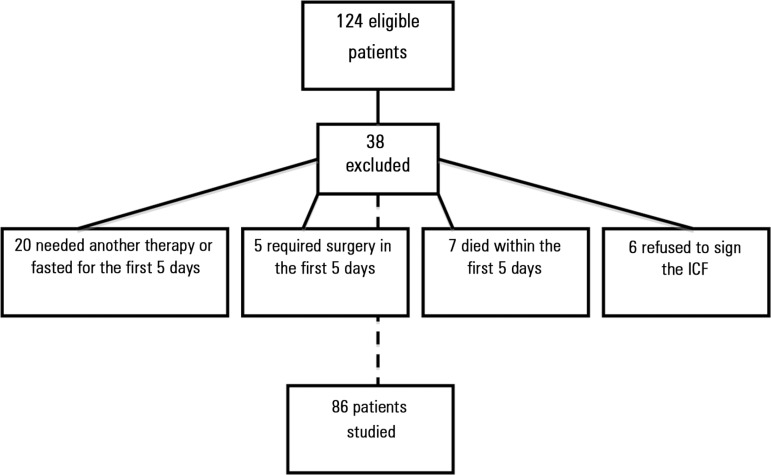
Total number of patients eligible and excluded from the study. ICF - informed consent form.

Of 124 eligible patients, 38 were excluded because of the need for another therapy, in addition to the enteral or fasting period, and because of hemodynamic instability, examinations or procedures in the first 5 days (20), surgical procedures (5), death (7) within the first 5 days, and family members not agreeing (6) to sign the ICF (Figure 1).

A total of 86 critical adult patients were prospectively studied, 43 (50%) of whom were hospitalized due to cardiorespiratory disease, 15 (17.5%) due to neurological conditions, 9 (10.5%) due to neoplasms, 8 (9.3%) due to trauma, such as from falling from the patient's own height, and 11 (12.7%) due to other causes.

The baseline characteristics of all patients and those who received > 35mL/kg or ≤ 35mL/kg of fluids are provided in table 1. Patients who received more than 35mL/kg of fluids had a lower estimated body weight (69.8 ± 15.3 *versus* 81.0 ± 5.3kg; p = 0.002), required more protein/kg (1.39 ± 0.16 *versus* 1.30 ± 0.18; p = 0.035) and showed a greater decrease in serum albumin levels (2.80 ± 0.50 *versus* 3.13 ± 0.60g/dL; p = 0.012) compared to those receiving ≤ 35mL/kg of fluids. There was no differences for the other data (p > 0.05).

**Table 1:** Baseline characteristics of all the studied patients and, among them, those who received > 35mL/kg or ≤ 35mL/kg of body weight of fluids on the first day

Variables	All	Infusion volume > 35mL/kg		*p value
	(n = 86)	Yes (n = 58)	No (n = 28)	
Age (years)	64.8 ± 17.4	64.4 ± 18.6	66.3 ± 13.1	0.582
Body weight (kg)	73.4 ± 16.2	69.8 ± 15.3	81.0 ± 5.3	0.002
SAPS III	64.6 ± 16.2	67.1 ± 17.5	57.7±10.1	0.165
Calorie needs (kcal)	24.14 ± 4.0	24.6 ± 4.1	23.2 ± 3.4	0.126
Protein requirements (g)	1.36 ± 0.17	1.39 ± 0.16	1.30 ± 0.18	0.035
Serum albumin (g/dL)	2.90 ± 0.56	2.80 ± 0.50	3.13 ± 0.60	0.012
CRP (mg/dL)	125.61 ± 83.6	125.7 ± 81.9	125 ± 88.6	0.982
CRP/albumin ratio	46.5 ± 34.0	48.4 ± 34.0	42.5 ± 34.0	0.464
Lactate (mmol/L)	23.7 ± 11.1	23.3 ± 11.1	24.7 ± 11.4	0.579
Blood sugar (mg/dL)	181.7 ± 50.5	176.0 ± 46.6	194.0 ± 57.2	0.133
Noradrenaline (mcg/kg/minute)	0.681 ± 0.678	0.729 ± 0.729	0.582 ± 0.555	0.350
Length of hospitalization (days)	32.2 ± 34.3	34.0 ± 39.01	28.4 ± 21.7	0.496
Elderly	63 (73.3)	42 (72.4)	21 (75)	0.800
Female sex	47 (54.7)	34 (58.6)	13 (46.4)	0.287
Nutritional status				
SGA = A†	9 (10.5)	5 (8.6)	4 (14.3)	0.421
SGA = B‡	64 (74.5)	42 (72.4)	22 (78.6)	0.540
SGA = C§	13 (15.1)	11 (19)	2 (7.1)	0.152
Mortality 28 in days	35 (40.7)	26 (44.8)	9 (32.1)	0.262

SAPS III - Simplified Acute Physiology Score III; CRP - C-reactive protein.

* p compares variables in relation to infusion volume > 35mL/kg or ≤ 35mL/kg;

† well-nourished;

‡ risk of moderate undernutrition or malnutrition;

§ severe malnutrition. Results are expressed as the mean ± standard deviation (Student’s *t*-test) or n (%) (chi-square test).

### Digestive tract disorders

ENT was started within 24 hours for all patients. The disorders of the digestive tract and volume drained by NG tube distributed to all patients and to those who received intravenous fluids > or ≤ 35mL/kg on the first day are provided in table 2. There was no difference between the groups (p > 0.05).

**Table 2:** Presence of digestive tract disorders, over 5 days, considering all patients and, among them, those who received > 35mL/kg or ≤ 35mL/kg of body weight of fluid on the first day

Variables	All	Infusion volume > 35mL/kg		*p value
	(n = 86)	Yes (n = 58)	No (n = 28)	
Constipation	64 (74.4)	42/58 (72.4%)	22/28 (78.6%)	0.854
Diarrhea	10 (11.6)	7/58 (12%)	3/28 (10.7%)	0.540
Abdominal distension	20 (23.3)	17/58 (29.3%)	3/28 (10.7%)	0.056
Melena	2 (2.3)	2/58 (3.4%)	0/28 (0%)	0.320
Drainage in NG tube (mL)	505 ± 412	513 ± 440	460 ± 251	0.842

NG tube - nasogastric tube.

* p compares variables in relation to infusion volume > 35 mL/kg or ≤ 35 mL/kg. Results are expressed as n (%) (chi-square test) or mean ± standard deviation (Student’s *t*-test).

### Infusion of intravenous fluids and sodium on the first day

On the first day of hospitalization, 58 (67.4%) patients received more than 35mL/kg of fluids, and 28 (32.6%) received ≤ 35mL/kg of body weight. The amount of intravenous fluids administered on the first day was 3,393.7 ± 1,417.0mL, corresponding to 48.2 ± 23.0 mL/kg, with a median of 44.4 (16.1 - 168.7) mL/kg. Sixty-five (75.6%) patients received a quantity of sodium ≥ 8.73g on the first day (cutoff in the 25th percentile). The mean infusion of sodium on the first day was 12.21 ± 5.1g, with a median of 10.8 (4.3 - 33.4) g.

### Intravenous fluids

Patients who received > 35mL/kg intravenous fluids on the first day received a lower volume of enteral diet over 5 days (2.781 ± 1.338mL *versus* 3.433 ± 1.202mL; p = 0.032) and a lower infused percentage (64.3 ± 19.7% *versus* 74.9 ± 18.6%; p = 0.020) than did those who received ≤ 35mL/kg. There were no differences for the other nutritional variables studied (p > 0.05). These results are provided in table 3. The number of patients receiving ENT with volumes below 2,063mL over 5 days (cutoff point, 25th percentile) was approximately 4.5 times higher in the group with the highest intravenous fluid infusion (19/58 (32.7%) *versus* 2/28 (7.1%); p = 0.010).

**Table 3:** Prescribed and infused enteral diet and protein-calorie deficiency, considering all patients and, among them, those who received > 35mL/kg or ≤ 35mL/kg of body weight of fluid on the first day

Variables	All	Infusion volume> 35mL/kg		*p value
	(n = 86)	Yes (n = 58)	No (n = 28)	
ENT prescribed 5 days (mL)	4,297 ± 1202	4,163 ± 1,245	4,573 ± 1,068	0.140
ENT infused 5 days (mL)	2,994 ± 1324	2,781 ± 1,338	3,433 ± 1,202	0.032
ENT prescribed on day 1 (mL)	553 ± 138	547 ± 139	565 ± 137	0.577
ENT infused on day 1 (mL)	185 ± 197	179 ± 205	196 ± 182	0.717
ENT infused 5 days (%)	67.8 ± 19.8	64.3 ± 19.7	74.9 ± 18.6	0.020
Caloric deficit of 5 days (kcal)	1813 ± 850	1868 ± 870	1699 ± 811	0.392
Protein Deficit of 5 days (g)	94.7 ± 46	95.9 ± 46.3	92.1 ± 45.9	0.722

ENT - enteral nutritional therapy.

* p compares variables in relation to infusion volume > 35mL/kg or ≤ 35mL/kg. Results are expressed as the mean ± standard deviation (Student’s *t*-test).

### Sodium

Patients who received intravenous infusion ≥ 8.73g sodium on the first day (cutoff point, 25th percentile) received a lower volume of enteral diet over 5 days (2,827 ± 1,397mL *versus* 3.509 ± 911.9mL; p = 0.013), had a lower percentage of infused diet (64.6 ± 19.5% *versus* 77.6 ± 17.9%; p = 0.008), and had a higher protein deficit (100 ± 49.3 *versus* 78 ± 28.5g; p = 0.014) than did those who received less than 8.73g of sodium on the first day (Table 4).

**Table 4:** Prescribed and infused enteral diet and protein-calorie deficiency, considering all patients and, among them, those that received sodium ≥ 8.73g on the first day

Variables	Sodium ≥ 8.73g		*p value
	Yes (n = 65)	No (n = 21)	
ENT prescribed 5 days (mL)	4,203 ± 1,265	4,586 ± 948	0.207
ENT infused 5 days (mL)	2,827 ± 1,397	3,509 ± 911	0.013
ENT prescribed on day 1 (mL)	540 ± 134	590 ± 144	0.149
ENT infused on day 1 (mL)	180 ± 205	198 ± 175	0.727
ENT infused 5 days (%)	64.6 ± 19.5	77.6 ± 17.9	0.008
Caloric deficit of 5 days (Kcal)	1,903 ± 879	1,532 ± 700	0.082
Protein deficit of 5 days (g)	100 ± 49.3	78 ± 28.5	0.014

ENT - enteral nutritional therapy. Results are expressed as the mean ± standard deviation (Student’s *t*-test).

## DISCUSSION

The findings showed that the administration of intravenous fluids on the first day of hospitalization at a volume greater than 35mL/kg may hinder enteral diet infusion. In this study, the mean fluid administered was approximately 48mL/kg, and some patients received almost 170mL/kg on the first day; some patients received almost 5 times the cutoff amount of 35mL/kg. The amount of sodium infused was also above the recommended cutoff amount, which is 2.0 g/day;^(25)^ some patients received 15 times this value. In addition to being rich in sodium, 0.9% saline solution is considered nonphysiological.^(26)^ According to Lobo et al.,^(16)^ patients who received a volume of saline solution ≥ 3.0L/day remained hospitalized longer and showed a delayed return of intestinal function, which may contribute to lower tolerance of enteral nutrition. Thus, although time is crucial in the resuscitation phase to achieve hemodynamic stability with the administration of fluids, in the first 3 hours, this overload can result in greater intestinal dysmotility.^(27)^ Positive fluid balance, intestinal loop edema, vomiting, gastroparesis and adynamic ileal are some of these complications.^(11,12,14,15,28)^ Alsous et al.^(29)^ showed that patients in septic shock with at least 1 day of negative water balance > 500mL in the first 3 days had lower mortality. Excess fluid can also cause an increase in body weight, from 3.0 to 7.0kg, which is associated with worse outcomes - mainly lower oxygen saturation and complications with surgical wounds.^(30)^ A recent study conducted with surgical patients showed a higher rate of infection at the surgical site and a higher risk of kidney injury, with no difference in septic complications and mortality between the restrictive group and liberal group. Although this study found benefits in the use of a more liberal volume, several others found results that favored patients who received a more restrictive volume of fluids.^(31)^ A study showed that the lower the intravenous fluid administration is, the greater the food intake. Patients who received less fluids (2.0L) and sodium (70mmol) per day had better gastric emptying and "willingness to feed", while the other group, which received a greater fluid volume, showed delayed gastric emptying and vomiting and did not feed normally.^(28)^ Additionally, in this study, patients from the restrictive group had earlier bowel recurrence and were discharged earlier.^(28)^ There is a close relationship between intestinal flow and the digestion and absorption of food;^(16)^ therefore, there are benefits of a more restrictive fluid and sodium protocol. Success in nutrition is related to lower fluid accumulation in the interstitium and greater weight loss due to increased fluid excretion, with consequent improvement of serum albumin.^(32)^ Thus, more restrictive fluid and sodium protocols may favor early oral/enteral nutrition.^(33)^ The replacement and administration of sodium and fluids should be addressed with careful planning so that better clinical outcomes are achieved.^(16)^ In the resuscitation phase, stability may be achieved with a lower intravenous fluid load in the presence of vasoactive drugs.^(22.23)^ Our data showed that intravenous fluid and sodium overload may have contributed, in some way, to lower enteral diet infusion, which resulted in caloric and protein deficits. This deficit in the first days is not compensated in the days following the hospitalization of critically ill patients.^(18)^ Critical protein-calorie deficiency in the ICU is approximately 70% and is associated with a lower probability of accumulated survival.^(34)^ A study by our group showed that increased fluid administration is associated with lower enteral diet infusion and protein-calorie deficiency in patients in intensive care.^(35)^ Several other studies have shown that the reduced supply of calories and protein can increase infectious complications, hospitalization stays, and mortality.^(36-38)^

Another important context is tolerance to nutritional therapy and the digestive tract. Among the factors that interfere with diet tolerance are gastrointestinal disorders and diet composition, but these are not the only ones.^(39,40)^ Our data showed that infusion of intravenous fluids > 35mL/kg contributed to lower infusion of enteral nutrition over 5 days. In our study, we found no difference in the amount of norepinephrine administered or in the SAPS III score for patients who received more or less than 35mL/kg. However, this study has a small number of cases, and thus, other multivariate analysis studies are needed to better study this relationship between the volume of intravenous fluids and the efficacy of enteral diet infusion in critically ill patients. Although this study has limitations, its findings may corroborate the development of more restrictive intravenous fluid administration protocols during the resuscitation phase. This more restrictive volume can result in better tolerance of an enteral diet as well as contribute to greater infusion of the diet with a subsequent reduction in calorie and protein deficits.

## CONCLUSION

The results of this study support the assumption that the administration of intravenous fluids, > 35mL/kg and sodium ≥ 8.73g, on the first day of hospitalization contributes to lower infusion of enteral diets in nonsurgical intensive care patients on mechanical ventilation.

## References

[1] Vincent JL, De Backer D, Wiedermann CJ (2016). Fluid management in sepsis: The potential beneficial effects of
albumin. J Crit Care.

[2] Silversides JA, Major E, Ferguson AJ, Mann EE, McAuley DF, Marshall JC (2017). Conservative fluid management or deresuscitation for patients
with sepsis or acute respiratory distress syndrome following the
resuscitation phase of critical illness: a systematic review and
meta-analysis. Intensive Care Med.

[3] Angus DC, van der Poll T (2013). Severe sepsis and septic shock. N Engl J Med.

[4] Zanotti-Cavazzoni SL, Guglielmi M, Parrillo JE, Walker T, Dellinger RP, Hollenberg SM (2009). Fluid resuscitation influences cardiovascular performance and
mortality in a murine model of sepsis. Intensive Care Med.

[5] Boyd JH, Forbes J, Nakada TA, Walley KR, Russell JA (2011). Fluid resuscitation in septic shock: a positive fluid balance and
elevated central venous pressure are associated with increased
mortality. Crit Care Med.

[6] Raghunathan K, Murray PT, Beattie WS, Lobo DN, Myburgh J, Sladen R, Kellum JA, Mythen MG, Shaw AD, ADQI XII Investigators Group (2014). Choice of fluid in acute illness: what should be given? An
international consensus. Br J Anaesth.

[7] Byrne L, Obonyo NG, Diab SD, Dunster KR, Passmore MR, Boon AC (2018). Unintended consequences: fluid resuscitation worsens shock in an
ovine model of endotoxemia. Am J Respir Crit Care Med.

[8] Sánchez M, Jiménez-Lendínez M, Cidoncha M, Asensio MJ, Herrerot E, Collado A (2011). Comparison of fluid compartments and fluid responsiveness in
septic and non-septic patients. Anaesth Intensive Care.

[9] Prowle JR, Echeverri JE, Ligabo EV, Ronco C, Bellomo R (2010). Fluid balance and acute kidney injury. Nat Rev Nephrol.

[10] Rehm M, Hulde N, Kammerer T, Meidert AS, Hofmann-Kiefer K (2017). State of the art in fluid and volume therapy: A user-friendly
staged concept. Anaesthesist.

[11] Rosenberg AL, Dechert RE, Park PK, Bartlett RH, NIH NHLBI ARDS Network (2009). Review of a large clinical series: association of cumulative
fluid balance on outcome in acute lung injury: a retrospective review of the
ARDS net tidal volume study cohort. J Intensive Care Med.

[12] Boyd JH, Forbes J, Nakada TA, Walley KR, Russell JA (2011). Fluid resuscitation in septic shock: a positive fluid balance and
elevated central venous pressure are associated with increased
mortality. Crit Care Med.

[13] Payen D, De Pont AC, Sakr Y, Spies C, Reinhart K, Vincent JL, Sepsis Occurrence in Acutely Ill Patients (SOAP)
Investigators (2008). A positive fluid balance is associated with a worse outcome in
patients with acute renal failure. Crit Care.

[14] Gong YC, Liu JT, Ma PL (2018). Early fluid loading for septic patients: Any safety limit
needed?. Chin J Traumatol.

[15] Hoste EA, Maitland K, Brudney CS, Mehta R, Vincent JL, Yates D, Kellum JA, Mythen MG, Shaw AD, ADQI XII Investigators Group (2014). Four phases of intravenous fluid therapy: a conceptual
model. Br J Anaesth.

[16] Lobo DN (2004). Fluid, eletrolytes and nutrition: physiological and clinical
aspects. Proc Nutr Soc.

[17] Kreymann KG, Berger MM, Deutz NE, Hiesmayr M, Jolliet P, Kazandjiev G, Nitenberg G, van den Berghe G, Wernerman J, Ebner C, Hartl W, Heymann C, Spies C, DGEM (German Society for Nutritional Medicine), ESPEN (European Society for Parenteral and Enteral
Nutrition) (2006). ESPEN Guidelines on Enteral Nutrition: Intensive
care. Clin Nutr.

[18] Villet S, Chiolero RL, Bollmann MD, Revelly JP, Cayeux R N MC, Delarue J (2005). Negative impact of hypocaloric feeding and energy balance on
clinical outcome in ICU patients. Clin Nutr.

[19] Heidegger CP, Darmon P, Pichard C (2008). Enteral vs. parenteral nutrition for the critically ill patient:
a combined support should be preferred. Curr Opin Crit Care.

[20] Chowdhury AH, Lobo DN (2011). Fluids and gastrointestinal function. Curr Opin Clin Nutr Metab Care.

[21] Peake SL, Delaney A, Bailey M, Bellomo R, Cameron PA, Cooper DJ, ARISE Investigators, ANZICS Clinical Trials Group (2014). Goal-directed resuscitation for patients with early septic
shock. N Engl J Med.

[22] Dellinger RP, Levy MM, Rhodes A, Annane D, Gerlach H, Opal SM, Sevransky JE, Sprung CL, Douglas IS, Jaeschke R, Osborn TM, Nunnally ME, Townsend SR, Reinhart K, Kleinpell RM, Angus DC, Deutschman CS, Machado FR, Rubenfeld GD, Webb S, Beale RJ, Vincent JL, Moreno R, Surviving Sepsis Campaign Guidelines Committee including The
Pediatric Subgroup (2013). Surviving Sepsis Campaign: international guidelines for
management of severe sepsis and septic shock, 2012. Intensive Care Med.

[23] Soni N (2009). British Consensus Guidelines on Intravenous Fluid Therapy for
Adult Surgical Patients (GIFTASUP): Cassandra's view. Anaesthesia.

[24] ASPEN Board of Directors and the Clinical Guidelines Task
Force (2002). Guidelines for the use of parenteral and enteral nutrition in
adult and pediatric patients. JPEN J Parenter Enteral Nutr.

[25] World Health Organization (WHO) (2006). Healthy ageing profiles. Guidance for producing local health profiles of
older people: report of OMS consultation.

[26] Allison S (2004). Fluid, electrolytes and nutrition. Clin Med (Lond).

[27] Lee SJ, Ramar K, Park JG, Gajic O, Li G, Kashyap R (2014). Increased fluid administration in the first three hours of sepsis
resuscitation is associated with reduced mortality: a retrospective cohort
study. Chest.

[28] Lobo DN, Bostock KA, Neal KR, Perkins AC, Rowlands BJ, Allison SP (2002). Effect of salt and water balance on recovery of gastrointestinal
function after elective colonic resection: a randomized controlled
trial. Lancet.

[29] Alsous F, Khamiees M, DeGirolamo A, Amoateng-Adjepong Y, Manthous CA (2000). Negative fluid balance predicts survival in patients with septic
shock: a retrospective pilot study. Chest.

[30] Brandstrup B, Tonnesen H, Beier-Holgersen R, Hjortso E, Ording H, Lindorff-Larsen K, Rasmussen MS, Lanng C, Wallin L, Iversen LH, Gramkow CS, Okholm M, Blemmer T, Svendsen PE, Rottensten HH, Thage B, Riis J, Jeppesen IS, Teilum D, Christensen AM, Graungaard B, Pott F, Danish Study Group on Perioperative Fluid Therapy (2003). Effects of intravenous fluid restriction on postoperative
complications: comparison of two perioperative fluid regimens: a randomized
assessor-blinded multicenter trial. Ann Surg.

[31] Myles PS, Bellomo R, Corcoran T, Forbes A, Peyton P, Story D, Christophi C, Leslie K, McGuinness S, Parke R, Serpell J, Chan MTV, Painter T, McCluskey S, Minto G, Wallace S, Australian and New Zealand College of Anaesthetists Clinical Trials
Network and the Australian and New Zealand Intensive Care Society
Clinical Trials Group (2018). Restrictive versus liberal fluid therapy for major abdominal
surgery. N Engl J Med.

[32] Lobo DN, Bjarnason K, Field J, Rowlands BJ, Allison SP (1999). Changes in weight, fluid balance and serum albumin in patients
referred for nutritional support. Clin Nutr.

[33] Selby LV, Rifkin MB, Yoon SS, Ariyan CE, Strong VE (2016). Decreased length of stay and earlier oral feeding associated with
standardized postoperative clinical care for total gastrectomies at a cancer
center. Surgery.

[34] Siqueira-Paese MC, Dock-Nascimento DB, De Aguilar-Nascimento JE (2016). Critical energy deﬁcit and mortality in critically ill
patients. Nutr Hosp.

[35] Arantes SS, Silva JM Jr, De Aguilar-Nascimento JE, Dock-Nascimiento DB (2018). Effects of intravenous fluid overload on caloric and protein
deficit in critically ill patients. Nutr Hosp.

[36] Rubinson L, Diette GB, Song X, Brower RG, Krishnan JA (2004). Low caloric intake is associated with nosocomial bloodstream
infections in patients in the medical intensive care unit. Crit Care Med.

[37] Singer P, Anbar R, Cohen J, Shapiro H, Shalita-Chesner M, Lev S (2011). The tight calorie control study (TICACOS): a prospective,
randomized, controlled pilot study of nutritional support in critically ill
patients. Intensive Care Med.

[38] Heidegger CP, Berger MM, Graf S, Zingg W, Darmon P, Costanza MC (2013). Optimisation of energy provision with supplemental parenteral
nutrition in critically ill patients: a randomised controlled clinical
trial. Lancet.

[39] De Jonghe B, Appere-De-Vechi C, Fournier M, Tran B, Merrer J, Melchior JC (2001). A prospective survey of nutritional support practices in
intensive care unit patients: what is prescribed? What is
delivered?. Crit Care Med.

[40] Lee ZY, Barakatun-Nisak MY, Noor Airini I, Heyland DK (2016). Enhanced Protein-Energy Provision via the Enteral Route in
Critically Ill Patients (PEP uP Protocol): A Review of
Evidence. Nutr Clin Pract.

